# Supplementary figures and images for: Oral Microbiome in Relation to Periodontitis Severity and Systemic Inflammation
Source: Int J Mol Sci. 2021 May 30;22(11):5876. doi: 10.3390/ijms22115876 (PMC8199296; doi:10.3390/ijms22115876)

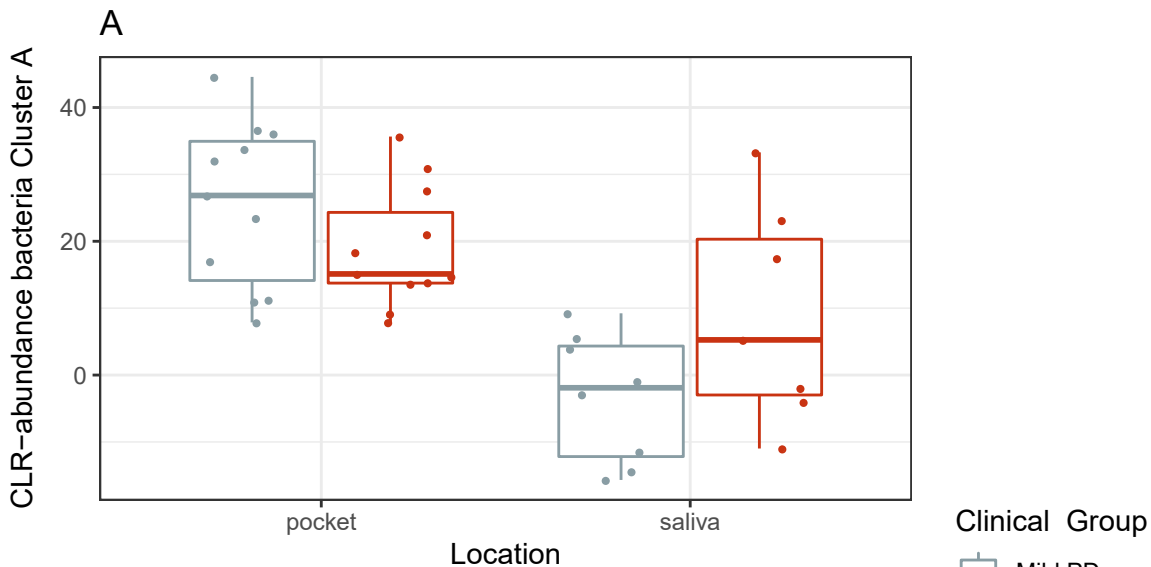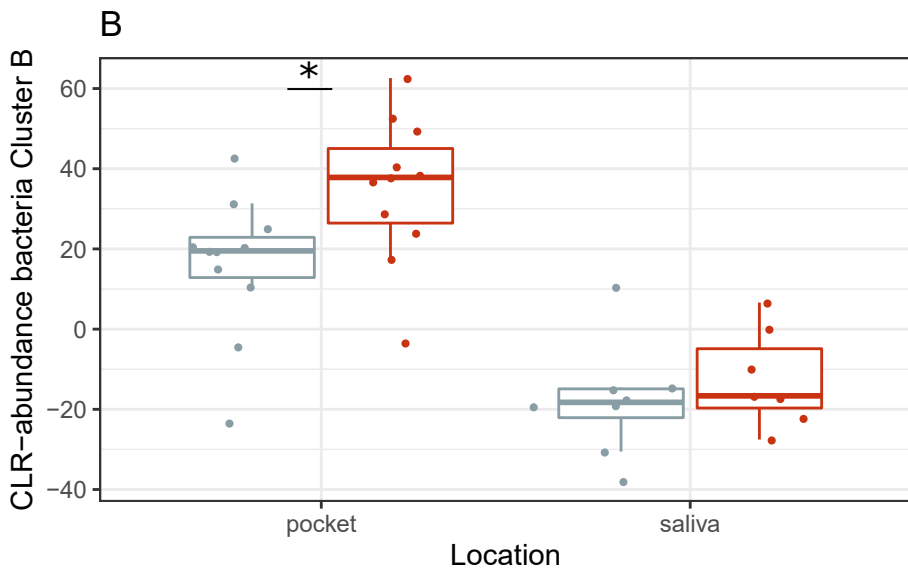

Supplement: Supplementary file 1 [file ijms-22-05876-s001.zip › FigureS1.pdf]

a

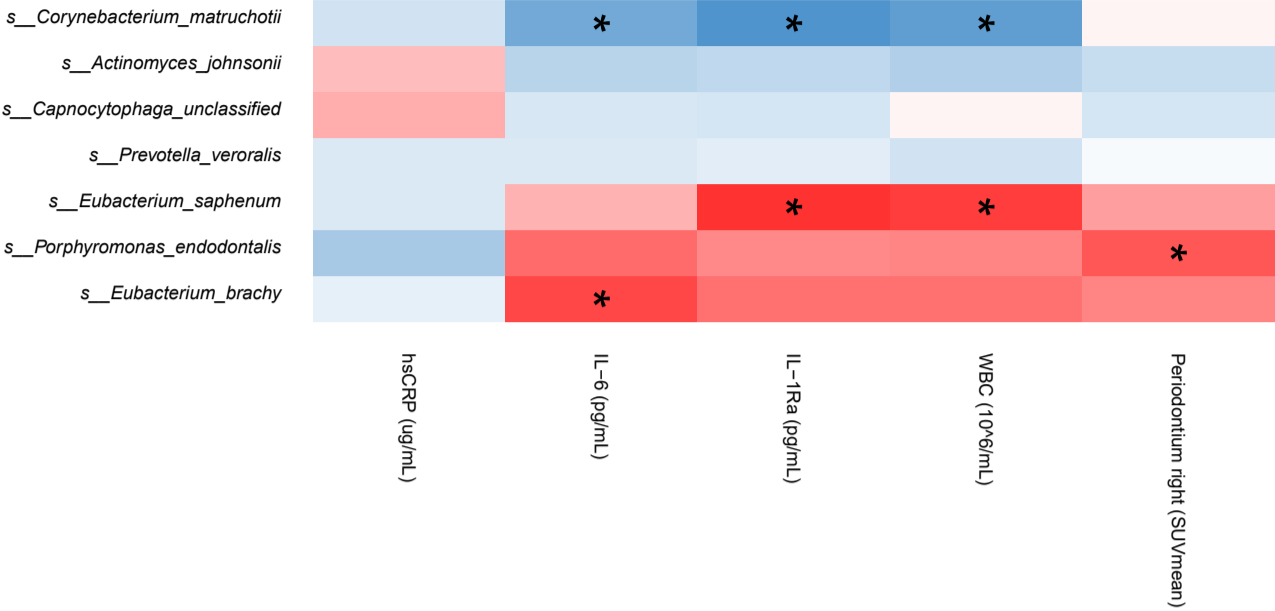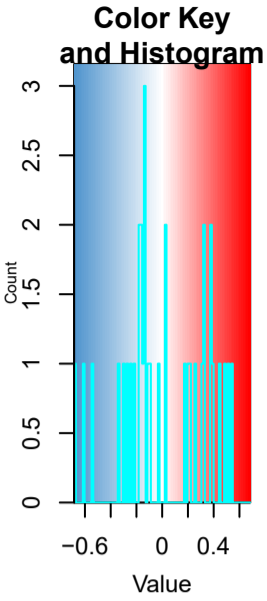

b

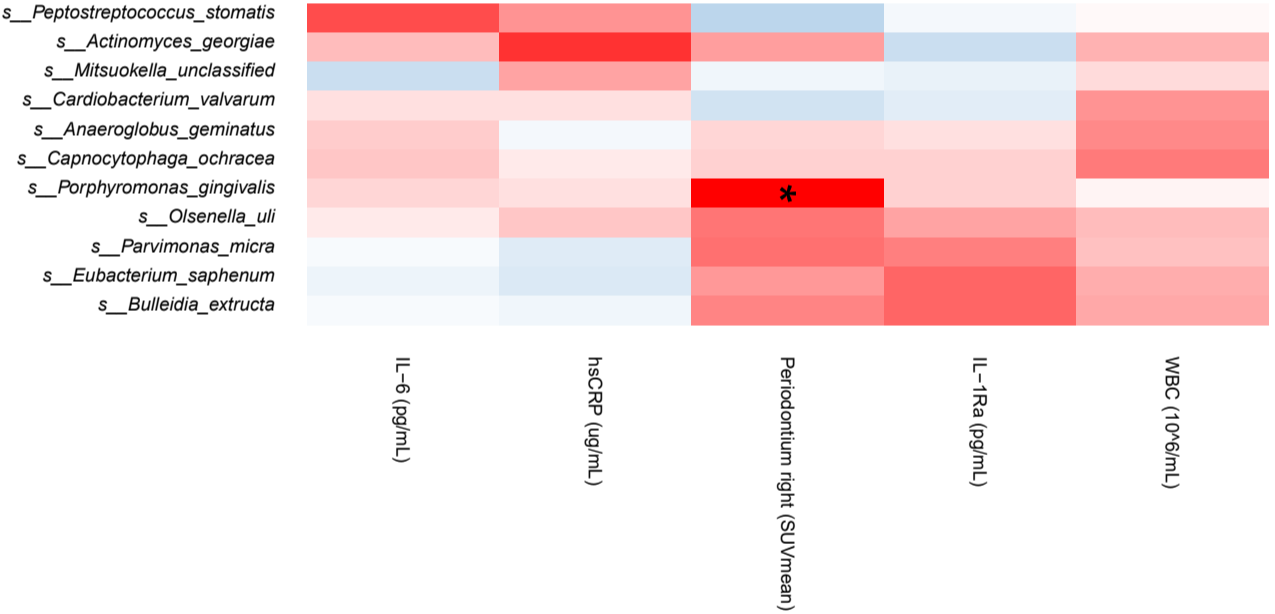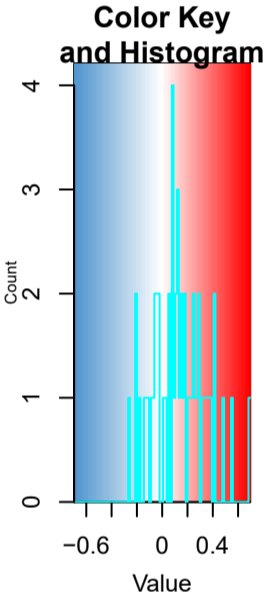

c

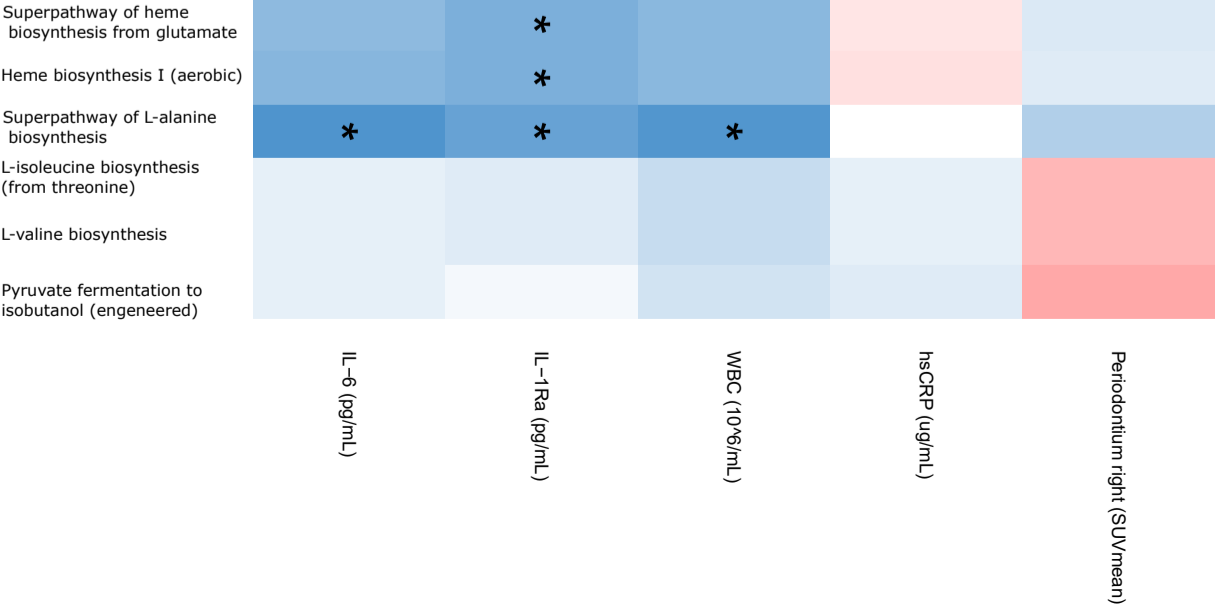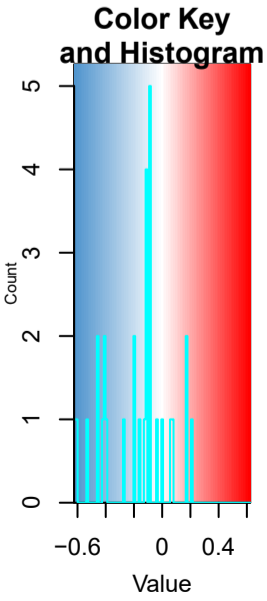

Supplement: Supplementary file 1 [file ijms-22-05876-s001.zip › FigureS2.pdf]
